# Influencing factors of self-perceived aging among empty nesters: a cross-sectional study

**DOI:** 10.15649/cuidarte.3500

**Published:** 2024-06-05

**Authors:** Shuxing Li, Yameng Dong, Xuebo Li

**Affiliations:** 1 University of Science and Technology, Tangshan, China. E-mail: 419154759@qq.com University of Science & Technology of China University of Science and Technology Tangshan China 419154759@qq.com; 2 Binzhou City People's Hospital. E-mail: lxb13700320080@163.com City People's Hospital Binzhou lxb13700320080@163.com; 3 University of Science and Technology, Tangshan, China. E-mail: 2305809688@qq.com University of Science & Technology of China University of Science and Technology Tangshan China 2305809688@qq.com

**Keywords:** Self Concept, Aging, Loneliness, Activities of Daily Living, Statistical Factor Analysis, Autoconcepto, Envejecimiento, Soledad, Actividades de la Vida Diaria, Análisis Factorial, Autoconceito, Envelhecimento, Solidáo, Atividades da Vida Diária, Análise Fatorial

## Abstract

**Introduction:**

The mental health issues faced by empty nesters deserve more attention. Studying self-perception of aging (SPA) and its influencing factors among empty nesters may help prevent negative SPA.

**Objective:**

To analyze situations and factors influencing SPA among elderly empty nesters, especially the mediating role of daily living ability and loneliness.

**Materials and Methods:**

A cross-sectional study was conducted in two communities in Binzhou City, Shandong Province of China. A total of 613 elderly empty nesters were studied using a self-designed general information questionnaire, the Brief Ageing Perception Questionnaire (B-APQ), the Activities of Daily Living (ADL) Scale, and the UCLA Loneliness Scale (ULS-6). Structural equation modeling (SEM) was used to analyze the relationships between variables.

**Results:**

Pathway analysis of SPA influencing factors shows that marital status, children’s visits, and self reported health had an indirect effect on SPA through loneliness. Chronic disease status had an indirect effect on SPA through daily living ability. Time outdoors had an indirect effect on SPA through activities of daily living and loneliness.

**Discussion:**

Daily life ability, chronic disease status, time outdoors, loneliness, self-reported health, marital status, and children's visits affected SPA among empty nesters.

**Conclusion:**

Empty nesters' SPA is relatively negative, and there are many influencing factors. It is important to improve empty nesters' abilities to manage healthcare tasks, increase outdoor activities and family members’ care, and provide comprehensive interventions to help them navigate aging.

## Resumen

**Introducción:**

Los problemas de salud mental que presentan las personas cuyos hijos ya no viven con ellas merecen mayor atención. El estudio de la autopercepción del envejecimiento y sus factores influyentes en esta población puede ayudar a la prevención de percepciones negativas del envejecimiento.

**Objetivo:**

Analizar las situaciones y factores influyentes en la autopercepción del envejecimiento de los adultos mayores cuyos hijos ya no viven con ellos, específicamente, el rol mediador de las actividades de la vida diaria y la soledad.

**Materiales y métodos:**

Se realizó un estudio transversal en 2 comunidades de la ciudad de Binzhou, provincia de Shandong, China. Se estudiaron 613 adultos mayores cuyos hijos ya no vivían con ellos, aplicando un cuestionario de información general, el Cuestionario Breve de Percepción del Envejecimiento (B-APQ), la Escala de Actividades de la Vida Diaria (ADL) y la Escala de Soledad de la Universidad de California (ULS-6). Se utilizaron modelos de ecuaciones estructurales para analizar las relaciones entre las variables.

**Resultados:**

El análisis de los factores influyentes en el envejecimiento autopercibido muestra que el estado civil, las visitas de los hijos y la soledad afectan de manera indirecta la autopercepción del envejecimiento. Asimismo, las enfermedades crónicas y la capacidad para realizar las actividades de la vida diaria afectan de manera indirecta la autopercepción del envejecimiento.

**Discusión:**

La capacidad para realizar actividades de la vida diaria, las enfermedades crónicas, salir, la soledad, el estado de salud autoreportado, el estado civil y las visitas de los hijos son algunos factores que influyen en la autopercepción del envejecimiento en esta población.

**Conclusión:**

La autopercepción del envejecimiento de las personas cuyos hijos ya no viven con ellas es relativamente negativa y hay muchos factores influyentes. Es importante mejorar el manejo que se le da a la salud de esta población, aumentando las actividades al aire libre y el cuidado de sus familiares e implementando intervenciones integrales para ayudarlos en el tratamiento del envejecimiento.

## Resumo

**Introdujo:**

Os problemas de saúde mental dos ninhos vazios merecem maior atenção. Estudar a autopercepção do envelhecimento da situado e os fatores de influencia entre os ninhos vazios ajudaria os ninhos vazios a prevenirem a autopercepção negativa do envelhecimento.

**Objetivo:**

Analisar a situado e os fatores de influencia da autopercepção do envelhecimento entre idosos de ninhos vazios, explicitando o papel mediador da capacidade de vida diária e da solidáo nos fatores de influencia da autopercepção do envelhecimento.

**Materiais e Métodos:**

Um estudo transversal foi realizado em 2 comunidades na cidade de Binzhou, na província de Shandong. 613 idosos de ninhos vazios foram pesquisados usando um questionário de informales gerais auto elaborado, Brief Aging Perceptions Questionnaire (B-APQ), Activities of Daily Living (ADL), Loneliness Scale-6 (ULS-6) e modelagem de equa^óes estruturais foram usadas para analisar as relajees entre as variáveis.

**Resultados:**

A análise do caminho da autopercepção do envelhecimento dos fatores que influenciam mostra: estado civil, filhos para visitar, autorrelato de saúde por meio da solidáo tiveram efeito indireto na autopercep^áo do envelhecimento. O status de doenças crónicas através da capacidade de vida diária teve efeito indireto na autopercepção do envelhecimento. Sair de casa devido a capacidade de vida diária e a solidáo teve efeito indireto na autopercepção do envelhecimento.

**Discussáo:**

O efeito da capacidade de vida diária, doenças crónicas, saídas, solidáo, autorrelato de saúde, estado civil e visita de crianzas na autopercepção do envelhecimento entre pessoas com o ninho vazio.

**Conclusáo:**

A autopercepção do envelhecimento dos ninhos vazios é relativamente negativa e há muitos fatores que influenciam. É importante melhorar a capacidade de gestáo da saúde dos ninhos vazios, aumentar as atividades ao ar livre e o cuidado dos familiares, e adotar medidas de intervengo abrangentes para ajudá-los a tratar o envelhecimento.

## Introduction

Self-perception of aging (SPA) refers to the subjective perception and emotional response experienced by older individuals when facing physical, psychological, social, and other challenges inherent in the aging process^1^. SPA can be categorized into two types: positive SPA and negative SPA. Positive SPA encompasses positive experiences, such as the accumulation of knowledge with age, while negative SPA involves negative experiences, such as declining physical and mental function associated with aging^2^. Positive SPA aids older adults in correctly understanding the aging process and extending their lifespan^3^. Conversely, negative SPA predicts a pessimistic mindset in older adults over the next five years. Negative SPA not only impacts physical and mental well-being and potentially triggers a "self-fulfilling" prophecy effect^4^ in this population, but it is also considered a stressor, leading to a series of stressful events in older individuals^5^.

Furthermore, SPA is considered a significant predictor of health and longevity^6^. Levy et al.’s^7^ study on aging showed that older individuals with a negative SPA lived 7.5 years fewer than those with a more positive SPA. Such impact of SPA on mortality, even in very old participants (over 78 years), was confirmed^8^. A 16-year longitudinal study involving 1507 older Australians, aged 65 to 103 years at baseline, showed a stronger association between baseline SPA levels and mortality; for a one-point increase in SPA baseline scores, there was a 12% higher risk of death over a 16-year period^9^. Research involving 140 individuals aged 65 years and older who were recently diagnosed with nonmetastatic cancer (breast, lung, gynecological, or hematological cancers) found that individuals with higher levels of negative SPA were 3.62 times more likely to die than those with a more positive SPA^10^. In addition, SPA independently and significantly predicts various aspects of physical function in older individuals, including walking speed^11^, cognitive function^12^, and quality of life^13^. Besides, how individuals perceive and respond to SPA can predict changes in the health trajectory of older persons, such as chronic diseases, physical disabilities, and frailty^14^.

Theories of aging suggest that older people often narrow their social circles compared to other age groups (such as adults, teenagers, or children) and pay more attention to their physical and psychological health. Empty nesters are older individuals who live alone or with a spouse. Because they lack childcare and emotional support from their children, empty nesters are more susceptible to feelings of loneliness^15^. Loneliness poses a significant challenge for the empty-nest elderly. A Chinese study conducted in Tangshan, which involved 2529 urban empty nesters aged over 60, demonstrated that all participants were experiencing loneliness feelings^16^. In another study conducted in China, 665 urban empty nesters over 60 years in Chifeng were found to experience more serious loneliness compared to their non-empty-nesters counterparts^17^.

Many empty nesters also experience difficulties in activities of daily living. Research has demonstrated that between 42.3% and 60.5% of empty nesters face challenges in daily living tasks^18^. As their ability to perform activities of daily living declines, the subjective well-being of empty nesters decreases^19^. Several studies have indicated that maintaining good daily living abilities is associated with lower depression levels^20^. Additionally, both social inactivity and self-reported loneliness have been identified as independent risk factors for mortality^21^. However, fewer studies have analyzed the relationship between daily living ability, loneliness, and SPA compared to those examining the outcomes of SPA^22^.

To prevent negative SPA, it is essential to identify the factors contributing to its development. This study aims to address the following research questions:

Question 1. What is the prevalence of SPA among empty-nest older adults in China?
Question 2. Do daily living abilities and loneliness directly affect empty nesters' SPA?
Question 3. Do daily living abilities and loneliness play an important role in the factors that influence empty nesters' SPA?

## Materials and Methods

### Study design

A single-center cross-sectional study was conducted. The EQUATOR network was consulted to identify reporting guidelines aimed at enhancing the reporting quality of observational epidemiological studies.

**Figure 1:**
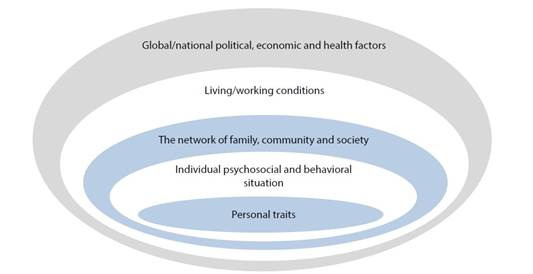
Social Ecological Model

Hypothesis: The study builds upon a theoretical hypothesis drawn from previous regression analyses and McLeroy’s Social Ecological Model for health promotion^23^ to understand empty nesters’ SPA, as shown in Figure 1. We hypothesized that SPA is a multidimensional phenomenon influenced by various factors related to participants' physio-psychosocial situation, such as sociodemographic characteristics, activity, relatedness category, family support, loneliness, and others.

### Setting

**Figure 2:**
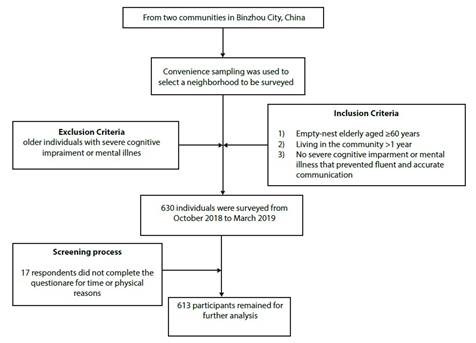
Participants' selection flow diagram

To obtain a representative sample, we employed a convenience sampling method. Researchers were selected as samples through random encounters in the community. Two communities in Binzhou City were randomly chosen. Upon obtaining informed consent from the communities, researchers conducted door-to-door surveys. Elderly empty nesters who meet the criteria within a community in Binzhou City were selected as the research subjects. Researchers recorded the location of specific families for differentiation purposes. Figure 2shows a flow diagram detailing the participant selection process.

Participants

We conducted a survey of 630 residents from two communities in Binzhou City, China (Figure 1). Inclusion criteria were as follows: 1) empty nesters aged > 60 years; 2) permanent residents who have lived in the community for more than one year; 3) no severe cognitive impairment or mental illness, allowing for fluent and accurate communication. Exclusion criteria comprised severe cognitive impairment or mental illness. Of the total 630 questionnaires distributed, 613 were effectively received, yielding an effective response rate of 97.30%. Seventeen questionnaires were withdrawn midway through the survey because respondents could not complete them for time or physical reasons.

### Data collection

Data collection took place from October 2018 to March 2019. Two graduate students were rigorously trained, and questionnaires were administered only after obtaining informed consent from the respondents. Ethical approval for the study was obtained from the North China University of Science and Technology. Unified guidelines for face-to-face, one-to-one questionnaire surveys were followed, allowing the respondents to fill out the form or answer the questions themselves. Questionnaires were completed and collected on-site. Subsequently, each was numbered individually, and all were carefully reviewed. To ensure data accuracy and completeness, we adhere to the principle of double entry for questionnaire data, which was meticulously cross-checked.

### Measurements

The *General Information Questionnaire* allowed us to gather sociodemographic data and relatedness variables likely to influence older adults' SPA. For data collection, we used a self-reported interview questionnaire comprising three sections. The sociodemographic section gathered information such as sex, age, and marital status. The family relationship section covers aspects like the number of children and how often they visit. The personal health status section covered aspects like daily living ability, chronic disease status, time outdoors, loneliness, and self-reported health. The participants rated the variables using a 4-point Likert scale.

The Brief Ageing Perception Questionnaire (B-APQ)^24^ consists of 17 items across five dimensions: Time-line chronic, consequences-positive, control-positive, consequences and control-negative, and emotional representations. It demonstrated strong internal consistency with a Cronbach's alpha of 0.914. The questionnaire uses a 5-point Likert scale ranging from 1 to 5, indicating responses from “strongly disagree” to “strongly agree”. The total score ranged from 17 to 85. A higher total score indicated a more negative SPA.

The Activity of Daily Living (ADL) Scale^25^ consists of 14 items that includes tasks like using public transport, eating, dressing, and walking. These items are not grouped by dimensions. A 4-point Likert scale is used to score from 1 to 4, where responses correspond to “I can do it myself” to “I can't do it at all.” The total score ranges from 14 to 56. A total score of 14 indicates complete normalcy. Respondents scoring between 14 and 22 were categorized as experiencing mild ADL disability, while those with a total score >22 were classified as having severe ADL disability.

The UCLA Loneliness Scale (ULS-6)^26^ contains six items and has a Cronbach's alpha of 0.87. The ULS- 6 uses a 4-point Likert scale ranging from 1 to 4, indicating responses from never to always. The total score ranges from 6 to 24. The higher the score on the ULS-6, the higher the level of loneliness experienced by the respondent.

### Statistical methods

Descriptive statistics were calculated to answer the first research question. Frequencies and percentages were used to analyze the sociodemographic characteristics and the prevalence of SPA. A test or analysis of variance was used to compare SPA prevalence across different characteristics or variables, addressing research question 2. To answer research question 3, structural equation modeling (SEM) was applied to observe the hypothesized relationships between variables and visually represent these relationships. The model's goodness of fit (GOF) was measured. The following values were adopted for an acceptable fit of the model: 1 < X2/DF < 3, RMSEA < 0.05, NFI > 0.90, TLI > 0.90, and CFI > 0.90^27^. If the GOF of the hypothesized model fell below the minimum requirements, a process of model modification was necessary to improve model fit. Once an emerging structural model was achieved, linear causal relationships between the domains (path coefficients and significance) and the indicators of model fit were examined: multivariate correlation coefficient (R2), standard errors (SE), and effect size (P). For p, commonality coefficients were interpreted as small when 0.02, average when 0.15, and significant when 0.35^28^. AMOS 24.0 software was used for SEM analysis. A p-value of < 0.05 was considered statistically significant. The data of the study are stored in Mendeley Data^29^.

### Ethical considerations

Guided by the Standards and Operational Guidance for the Ethical Review of Health-related Research with Human Participants, all the participants volunteered to participate in the present study. They had the right to withdraw at any time without any negative consequences. No names or locations were mentioned in the questionnaire. The University Research Ethics Committee on Human Research approved the required official permission for the present study (2019055).

## Results

### Characteristics

The total respondents in this study comprised 52.85% (324) males and 47.15% (289) females aged 60 to 89 (68.48±6.95). Regarding marital status, 90.04% (552) had a spouse, and 9.95% (61) had no spouse (divorced, unmarried, or widowed). Their chronic disease status was distributed as follows: 42.47% (262) had no chronic diseases, 35.88% (220) had one chronic disease, 16.47% (101) had two chronic diseases, 4.89% (30) had three or more chronic diseases. In terms of time outdoors, 9.79% (60) spent less than 1 hour a day outdoors, 17.46% (107) spent from 1 to 4 hours a day outdoors, 72.76% (446) spent more than 4 hours a day outdoors. Regarding self-reported health, 74.23% (455) reported good health, 16.97% (104) reported average health, and 8.81% (54) reported poor health. Regarding children’s visit frequency, 95.11% (583) received more visits, and 4.89% (30) received fewer visits.

### Self-perception of aging (SPA), daily living ability, and loneliness scores of empty nesters in the community

The community’s empty nesters’ had a B-APQ score of 41.36±5.98. The consequences and control negative dimension had the highest score, followed by the timeline-chronic dimension, as shown in Table 1. Additionally, there were 428 patients with normal daily living ability, 143 with mild disability, and 42 with severe disability. The ULS-6 score averaged 10.82±2.42.

**Table 1:** Scores among dimensions of the Brief Ageing Perception Questionnaire (B-APQ) in empty nesters

Dimensions	Score	Average
Timeline-chronic	10.08±2.47	3.36±0.82
Consequences-positive	4.43±1.34	2.48±0.45
Control-positive	3.43±1.46	2.14±0.49
Consequences and control-negative	16.40±2.69	3.28±0.54
Emotional representations	7.04±1.35	2.35±0.45
Total B-APQ score	41.36±5.98	2.79±0.35

### Relationships between empty nesters' SPA and sociodemographic characteristics

The SPA scores among empty nesters regarding marital status, chronic disease status, time outdoors, self-reported health, children's visits, daily living ability, and loneliness were statistically significant (p <0.01), as shown in Table 2. Correlation analysis revealed significant positive correlations between marital status, children's visits, chronic disease status, loneliness, and SPA (r=0.281, 0.143, 0.332, 0.177, p<0.01). Conversely, time outdoors, self-reported health, daily living ability, and SPA showed significant negative correlations (r=-0.341, -0.314, -0.500, p<0.01).

**Table 2:** Correlations between empty nesters' self-perception of aging (SPA) and sociodemographic characteristics

Characteristics	%(n) 613	Self-perception of aging score	F/t	P-value
Marital status			-7.236	<0.001
With a spouse	90.04(552)	40.80±5.73		
No spouse	9.95(61)	46.41±5.93		
Chronic diseases status			26.519	<0.001
0	42.47(262)	39.23±5.60		
1	35.88(220)	42.08±5.80		
2	16.47(101)	44.32±5.24		
>3	4.89(30)	44.73±6.10		
Time outdoors (hours per day)			42.355	<0.001
<1	9.79(60)	45.63±5.78		
>1 and <4	17.46(107)	44.21±5.94		
>4	72.76(446)	40.10±5.51		
Self-reported health			34.863	<0.001
Poor	8.81(54)	45.52±5.05		
Average	16.97(104)	44.05±4.88		
Good	72.76(455)	40.25±5.91		
Children's visit			2.473	0.014
More frequent	95.11(583)	41.49±5.93		
Less frequent	4.89(30)	38.73±6.43		
Daily living ability			105.784	<0.001
Normal	69.82(428)	39.41±5.31		
Mild disability	23.33(143)	45.20±4.89		
Severe disability	6.85(42)	48.10±4.49		
Loneliness			-4.454	<0.001
No	53.51(328)	40.37±5.95		
Yes	46.49(285)	42.49±5.82		

*P-value: Pearson's chi-squared test*

### The mediating role of daily living ability and loneliness: Structural equation modeling analysis

**Figure 3:**
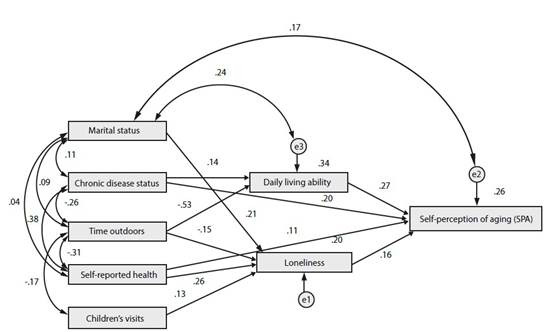
Pathway analysis of self-perception of aging (SPA) influencing factors in empty nesters

Based on the literature and objective knowledge, the significant variables identified in the single factor analysis were set up as hypothesis models. The structural equation model incorporated marital status, chronic disease status, time outdoors, self-reported health, and children's visits as exogenous variables, while daily living ability, loneliness, and SPA were designated as endogenous variables. After adjustments, this model demonstrated a good fitting degree (c2/df=2.365, RMSEA=0.047, NFI=0.975, TLI=0.954, and CFI=0.985). As shown in Table 3, daily living ability, chronic disease status, loneliness, and self-reported health directly affected SPA, with coefficients of 0.272, 0.198, 0.160, and 0.107, respectively. Additionally, chronic disease status, time outdoors, self-reported health, marital status, and children's visits indirectly affected SPA, with coefficients of 0.039, -0.167, 0.042, 0.034, and 0.021, respectively. Collectively, daily living ability, chronic disease status, time outdoors, loneliness, self reported health, marital status, and children’s visits all influenced empty nesters' SPA, either directly or indirectly, with total effects of 0.272, 0.237, -0.167, 0.160, 0.149, 0.034, and 0.021, respectively. The structural model shown in Figure 3 reveals that marital status, children's visits, and self-reported health indirectly affect SPA through loneliness, while chronic disease status indirectly affects SPA through daily living ability. Finally, time outdoors indirectly affects SPA through daily living ability and loneliness.

**Table 3:** Path analysis of factors influencing SPA of empty nesters with different characteristics

Variable	Direct effect	Indirect effect	Total effect	Effect of sorting
Daily living ability	0.272	---	0.272	1
Chronic disease status	0.198	0.039	0.237	2
Time outdoors	---	-0.167	-0.167	3
Loneliness	0.160	---	0.160	4
Self-reported health	0.107	0.042	0.149	5
Marital status	---	0.034	0.034	6
Children’s visit	---	0.021	0.021	7

## Discussion

### SPA status of empty nesters in the community

The results showed that the SPA score among empty nesters in the Binzhou community was 41.36±5.98. This score is lower when contrasted with the SPA score (69.0±16.0) reported for older individuals in the community by Zielińska-Więczkowska et al.^30^. This suggests that empty nesters exhibit a more negative SPA than older individuals in the community. The highest scores were observed in the timeline-chronic dimension and consequences and control-negative dimension, indicating that empty nesters perceive aging as an extended, periodic, and repeated process that is uncontrollable. Studies suggest that SPA plays a decisive role in the health outcomes of the older population^31^. Especially for empty nesters who lack a spouse, suffer from multiple chronic diseases, spend less time outdoors, report poor health, receive fewer visits from their children, experience poor daily living ability, and feel lonely, improving their SPA levels should be prioritized.

### Influencing factors of SPA among empty nesters in the community

The effect of daily living ability and chronic disease status on SPA empty nesters in the community were also examined. Daily living ability is an important indicator for evaluating the health status of older adults. The worse the daily living ability is, the worse the health status is^32^. Poor health often leads to negative SPA^33^. In this study, the direct effect of daily living ability on SPA was 0.272. The total effect of chronic disease status on SPA was 0.237, which directly and indirectly affects SPA through daily living ability. Empty nesters often coexist with a range of chronic diseases as their physical function declines with age. This decline in health status is frequently accompanied by negative psychological emotions, contributing to a more negative SPA. It is recommended that the community prioritize care for empty nesters with chronic diseases by increasing primary medical care and providing social and human care. Straightening their health management abilities is also crucial.

### Influence of time outdoors on SPA of empty nesters in the community

The results of this study show that time outdoors indirectly affects SPA through two mediating variables: daily living ability and loneliness, with a total effect of -0.167, which is an important protective factor in delaying SPA among empty nesters. Time outdoors includes engaging in outdoor physical exercise and recreational activities. Studies suggest that increasing outdoor time and engaging in moderate exercise can help maintain physical and mental health, indirectly improving daily life ability^34^. Furthermore, participating in recreational activities fosters new friendships and cultivates interests and hobbies, thereby improving older people's mental health, reducing loneliness and other negative emotions^35^, and fostering positive SPA. It is suggested that the community encourages empty nesters to engage in appropriate outdoor exercise, organizes more recreational and sports activities, and enriches their spiritual life.

### Effect of self-reported health and loneliness on SPA of empty nesters in the community

The results of this study showed that self-reported health had a total effect of 0.149 on SPA, affecting it not only directly but also indirectly through loneliness. Self-reported health serves as an important subjective measure of older individuals' health status and is an effective predictor of health outcomes^36^. Empty nesters who reported poor health are more likely to move slowly, stay at home, and reduce their engagement in social activities. Moreover, being away from their children can exacerbate feelings of loneliness among empty nesters. Prolonged loneliness can detrimentally affect their mental health and lead to a more negative SPA^37^. Therefore, it is recommended that the community prioritize support for empty nesters reporting poor health status, enhancing health education initiatives, and strengthening the community support system.

### Effect of marital status and frequency of children's visits on SPA of empty nesters the in community

The results of this study showed that both marital status and children's visits indirectly affect SPA through loneliness as a mediating variable, with effects of 0.034 and 0.021, respectively. Research suggests that older adults without spouses tend to experience higher levels of loneliness compared to those who have spouses. Additionally, empty nesters with spouses often engage in companionship and communication, which helps alleviate negative emotions^38^^, ^^39^. Frequent visits from their children provide empty nesters with opportunities to express their feelings opportunely, and respect and care from their children contribute to their sense of satisfaction, thus reducing negative emotions such as loneliness and fostering a more positive SPA. In light of this, it is recommended that the community promote a culture of remarriage, establish platforms for remarriage^40^, and encourage children to spend more time with their parents and provide increased family support.

## Conclusion

Daily living ability, chronic disease status, time outdoors, loneliness, self-reported health, marital status, and children's visits, directly and indirectly, affect empty nesters' SPA. To sum up, empty nesters in the community exhibit a negative SPA. Based on comprehensive consideration of their specific situation, it is essential to enhance empty nesters' abilities to manage healthcare tasks and increase opportunities for outdoor activities and family member care to help this population navigate the aging process positively and effectively.

### Limitations

The present study has some limitations. Firstly, both SPA and physical and mental health measures were self-reported, which could introduce response bias. Secondly, our study used a specific sample, thus the findings cannot be generalized to older adults from all regions. Future research should focus on exploring the full model of SPA, especially its applicability to other specific samples, such as the oldest age groups or non-empty nesters. These areas still remain to be explored in future studies.

### Implications to community practice

Community nurses are constantly challenged by how to ignite the vitality of empty nesters, encouraging them to live actively, exercise regularly to reduce disease and promote overall health. It is well known that maintaining a positive, youthful mindset plays a pivotal role in arousing people's enthusiasm. However, a negative SPA is undoubtedly a barrier to fostering a positive mindset among empty nesters. Our research has identified that daily living ability and loneliness are correlated factors contributing to negative SPA, serving as mediating variables among chronic disease status, time outdoors, self-reported health, marital status, children's visits, and negative SPA. Therefore, we recommend increasing both the frequency and duration of children's visits, organizing recreational activities, and exploring other ways to alleviate loneliness. These efforts can effectively mobilize empty nesters' initiative, ultimately improving their health and well-being.

## References

[1] Tovel H, Carmel S, Raveis VH (2019). Relationships Among Self-perception of Aging, Physical Functioning, and Self-efficacy in Late Life. J Gerontol B Psychol Sci Soc Sci.

[2] Cheng ST, Fung HH, Chan ACM (2009). Self-perception and psychological well-being: the benefits of foreseeing a worse future. Psychol Aging.

[3] Levy BR, Myers LM (2004). Preventive health behaviors influenced by self-perceptions of aging. Prev Med.

[4] Wurm S, Warner LM, Ziegelmann JP, Wolff JK, Schüz B (2013). How do negative self-perceptions of aging become a self-fulfilling prophecy?. Psychol Aging.

[5] Levy BR, Hausdorff JM, Hencke R, Wei JY (2000). Reducing cardiovascular stress with positive self stereotypes of aging. J Gerontol B Psychol Sci Soc Sci.

[6] Wurm S, Diehl M, Kornadt AE, Westerhof GJ, Wahl HW (2017). How do views on aging affect health outcomes in adulthood and late life? Explanations for an established connection. Dev Rev.

[7] Levy BR, Slade MD, Kunkel SR, Kasl SV (2002). Longevity increased by positive self-perceptions of aging. J Pers Soc Psychol.

[8] Zhang X, St Kamin, Liu S, Hh Fung, Lang FR (2020). Negative Self-perception of Aging and Mortality in Very Old Chinese Adults: The Mediation Role of Healthy Lifestyle. J Gerontol B Psychol Sci Soc Sci.

[9] Sargent-Cox KA, Anstey KJ, Luszcz MA (2014). Longitudinal change of self-perceptions of aging and mortality. J Gerontol B Psychol Sci Soc Sci.

[10] Schroyen S, Letenneur L, Missotten P, Jérusalem G, Adam S (2020). Impact of self-perception of aging on mortality of older patients in oncology. Cancer Med.

[11] Robertson DA, Savva GM, King-Kallimanis BL, Kenny RA (2015). Negative perceptions of aging and decline in walking speed: a self-fulfilling prophecy. PLoS One.

[12] Jianing Ma, Hong Sun, Lin Zhang, Lingdi Meng, Ting Jin, Leilei Guo (2023). Analysis of the correlation between self perceived aging and cognitive function in elderly patients with chronic diseases. Nursing practice and research.

[13] Yan Jiang, Jing Wu, Xue qin Zhou (2023). Effect of self-perceived aging and social support on quality of life in elderly patients with chronic diseases. J Practical Preventive Medicine.

[14] Feifei Wang, Guiying Yao, Xiuzhen Hou, Junjun Sun, Huimin Zhang, Hua Wang (2023). Mediating effects of self-perceptions of aging between frailty and cognitive function in community dwelling older adults. J Chinese Journal of Behavioral Medicine and Brain Science.

[15] Zuo Y, Tang M, Zhang YJ, Meng CX, Li CY, Sun Y (2022). Detection rate of anxiety symptoms of empty nesters in China: a meta-analysis. J Modern Preventive Medicine.

[16] Haoyan Zhang, Changxiang Chen, Min Zhang, Shuxing Li, Na Dou (2019). The influence factors of loneliness in empty-nest elderly people in Tangshan. J Chinese Journal of Gerontology.

[17] Hong Su, Yuqiu Zhou, Lina Wang, Mi Wang (2018). Loneliness status and influencing factors between urban empty nester and non-empty nest older adults. J Chinese Journal of Gerontology.

[18] Yanru Wang, Huiling Xia, Yongbing Liu (2014). The relationship between chronic disease and self care ability in daily life of empty-nest elderly in Urumqi. J Chinese Journal of Gerontology.

[19] Zhiyu Wang, Xiaojun Li, Qingfeng Ma, Ziyao Cheng (2016). Anxiety and depression affect subjective well-being in empty nesters. J Occupational and Health.

[20] Wenkai Y, Yang L (2020). Study on the correlation of depression, cognitive function and living ability in Chongqing, China. Alzheimer's & Dementia.

[21] Tilvis RS, Routasalo P, Karppinen H, Strandberg TE, Kautiainen H, Pitkala KH (2012). Social isolation, social activity and loneliness as survival indicators in old age; a nationwide survey with a 7-year follow-up. European Geriatric Medicine.

[22] Westerhof G, Wurm S (2015). Longitudinal Research on Subjective Aging, Health, and Longevity: Current Evidence and New Directions for Research. Annual Review of Gerontology and Geriatrics.

[23] McLeroy KR, Bibeau D, Steckler A, Glanz K (1988). An Ecological Perspective on Health Promotion Programs. Health Education Quarterly.

[24] Jaafar MH, Villiers-Tuthill A, Sim SH, Lim MA, Morgan K (2020). Validation of the Brief Ageing Perceptions Questionnaire (B-ApQ) in Malaysia. Aging & mental health.

[25] Wojtusiak J, Asadzadehzanjani N, Levy C, Alemi F, Williams AE (2021). Computational Barthel Index: an automated tool for assessing and predicting activities of daily living among nursing home patients. BMC Medical Informatics and Decision Making.

[26] Nazzal FI, Cruz O, Neto F (2018). Psychometric Analysis of the Short-Form UCLA Loneliness Scale (ULS- 6) Among Palestinian University Students. Interpersona.

[27] Byrne BM (2001). Structural Equation Modeling With AMOS: Basic Concepts, Applications, and Programming.

[28] Chen LP (2019). The statistical analysis of multivariate failure time data: A marginal modeling approach. Ross L. Prentice and Shanshan Zhao (2019). New York, NY. Biometrical Journal.

[29] Li S, Dong Y, Li X (2024). The influencing factors of self-perceived aging among empty nesters: a cross sectional study. Mendeley V1.

[30] Zielinska-Więczkowska H, Sas K (2020). The Sense of Coherence, Self-Perception of Aging and the Occurrence of Depression Among the Participants of the University of the Third Age Depending on Socio-Demographic Factors. Clin Interv Aging.

[31] Zhang W, Radhakrishnan K (2018). Evidence on selection, optimization, and compensation strategies to optimize aging with multiple chronic conditions: A literature review. Geriatr Nurs.

[32] Zhang Y, Xiong Y, Yu Q, Shen S, Chen L, Lei X (2021). The activity of daily living (ADL) subgroups and health impairment among Chinese elderly: a latent profile analysis. BMC Geriatr.

[33] Wurm S, Wolff JK, Schuz B (2014). Primary care supply moderates the impact of diseases on self -perceptions of aging. Psychol Aging.

[34] Salas-Salvado J, Diaz-Lopez A, Ruiz-Canela M, Basora J, Fito M, Corella D (2019). Effect of a Lifestyle Intervention Program With Energy-Restricted Mediterranean Diet and Exercise on Weight Loss and Cardiovascular Risk Factors: One-Year Results of the PREDIMED-Plus Trial. Diabetes Care.

[35] Guan J, Wang G, Geng C (2019). The Impact of Different Levels of Physical Activity on Health among Middle-Aged and Elderly Chinese Adults. Iran J Public Health.

[36] Saber M, Rashedi V, Fadakar-Davarani MM, Borhaninejad V (2021). Social Support, Happiness, and Self-Rated Health among Older Adults: A Population-Based Study. Advances in Gerontology.

[37] Diehl M, Wettstein M, Spuling SM, Wurm S (2021). Age-related change in self-perceptions of aging: Longitudinal trajectories and predictors of change. Psychol Aging.

[38] Gao F, Zhou L, Gao Y, Zhang Y, Zuo A, Zhang X (2022). Effects of physical and mental health factors and family function on the self-perception of aging in the elderly of Chinese community. Brain Behav.

[39] Cheng ST (2017). Self-Perception of Aging and Satisfaction With Children's Support. J Gerontol B Psychol Sci Soc Sci.

[40] Sherman CW, Webster NJ, Antonucci TC (2013). Dementia Caregiving in the Context of Late-Life Remarriage: Support Networks, Relationship Quality, and Well-being. Journal of Marriage and Family.

